# Osteomyelitis and Septic Arthritis Due to *Burkholderia pseudomallei*: A 10-Year Retrospective Melioidosis Study From South China

**DOI:** 10.3389/fcimb.2021.654745

**Published:** 2021-05-28

**Authors:** Hua Wu, Xuming Wang, Xiaojun Zhou, Shaowen Chen, Wenhui Mai, Hui Huang, Zelin You, Suling Zhang, Xiuxia Zhang, Binghuai Lu

**Affiliations:** ^1^ Department of Laboratory Medicine, Affiliated Hainan Hospital of Hainan Medical College, Haikou, China; ^2^ Department of Laboratory Medicine, Second Affiliated Hospital of Hainan Medical College, Haikou, China; ^3^ Department of Laboratory Medicine, Haikou Third People’s Hospital, Haikou, China; ^4^ Department of Laboratory Medicine, Affiliated Haikou Hospital of Xiangya Medical College, Central South University, Haikou, China; ^5^ Department of Laboratory Medicine, Ling Shui Li Autonomous County People’s Hospital, Lingshui, China; ^6^ Department of Laboratory Medicine, Danzhou People’s Hospital, Danzhou, China; ^7^ Department of Laboratory Medicine, The Second People’s Hospital of Ledong County, Ledong, China; ^8^ Laboratory of Clinical Microbiology and Infectious Diseases, Department of Pulmonary and Critical Care Medicine, China-Japan Friendship Hospital, National Clinical Research Center of Respiratory Diseases, Beijing, China

**Keywords:** *Burkholderia pseudomallei*, melioidosis, osteomyelitis, septic arthritis, bone and joint infection (BJI), antibiotic treatment

## Abstract

*Burkholderia pseudomallei* is the causative agent of melioidosis, endemic mainly in tropical and subtropical areas. Its clinical manifestation is broad ranging from a localized skin lesion to a life-threatening systemic disease. Osteomyelitis and septic arthritis caused by *B. pseudomallei* are a rare, fatal illness, whose clinical features have not been illustrated in mainland China. Over 10 years (2010 to 2019), of 334 culture-confirmed melioidosis in Hainan province, China, 44 patients (13.2%) were confirmed to have osteomyelitis and septic arthritis through the combination of clinical features, imaging examination and microbiological culture. Herein, we summarized these 44 patients’ clinical manifestations, demographical features, antibiotic treatment, and outcomes. Of them, osteomyelitis and septic arthritis accounted for 25 (56.8%) and 15 (34.1%), respectively, and 4 patients (9.1%) had both. The gender ratio of male/female was approximately 13.7:1; diabetes mellitus was the most common risk factor (38/44, 86.4%); imipenem and trimethoprim/sulfamethoxazole were the most frequently used antibiotics. Most *B. pseudomallei* strains were isolated from blood samples (41/44, 93.2%). After surgical handling, antibiotic treatment, or both, 9 patients died, with a mortality rate of 20.5%. In summary, in melioidosis endemic areas, for patients with both localized manifestations of joint and bone and a positive *B. pseudomallei* blood culture, increased awareness is required for melioidotic osteomyelitis and septic arthritis.

## Introduction

*Burkholderia pseudomallei* might cause melioidosis through inhalation or percutaneous inoculation, endemic in Southeast Asia and Northern Australia (Limmathurotsakul et al., 2016; Dance and Limmathurotsakul, 2018; Chakravorty A, 2019; Gassiep et al., 2020). In China, most melioidosis cases were reported in Hainan Province (Zheng et al., 2019; Wu et al., 2020; Zhu et al., 2020). *B. pseudomallei* can involve tissues and organs throughout the body and lead to variable clinical manifestations, ranging from mild localized abscess to invasive infections (Chanvitan et al., 2019; Gassiep et al., 2020; Wu et al., 2020). Bone and Joint Infection (BJI) caused by *B. pseudomallei*, including septic arthritis and osteomyelitis, is a rare but potentially severe infection which might end with bleak outcomes (Raja and Scarsbrook, 2016). Melioidosis lacks definite clinical symptoms with many of them shared similar manifestations with pyogenic bacterial infection and tuberculosis infection, making the diagnose of melioidosis challenging (Shenoy et al., 2009; Doker et al., 2014; Garg et al., 2020). The same is true for melioidotic osteomyelitis and septic arthritis. Empirical antibiotic therapy should be initiated as soon as possible after collecting appropriate samples for microbiologic tests in those patients suspected of having melioidosis with bone and joint involvement (Raja and Scarsbrook, 2016).

Melioidosis has been studied in mainland China over the past twenty years, but there is a limited description in the literature of the infection involving bone, joints, and soft tissues (Dong et al., 2018; Wu et al., 2019; Zheng et al., 2019; Wu et al., 2020; Zhu et al., 2020). Herein, 44 cases of melioidotic osteomyelitis and septic arthritis were reported with the aim of determining the prevalence of rheumatological involvement in melioidosis patients in China, and describing their clinical characteristics in terms of demographic and clinical profiles, treatment, and outcomes (Teparrakkul et al., 2008). This information should be useful to patients in melioidosis endemic countries (Weerachai Kosuwon, 2003; Raja and Scarsbrook, 2016).

## Materials and Methods

### Ethical Approval

The institutional review boards at Hainan General Hospital approved the study protocol. Individual consent was not sought from the patients involved as this was a retrospective study and focused only on the epidemical features of melioidosis osteomyelitis and septic arthritis, and the privacy of involved subjects was not affected.

### Biosafety Procedures

*B. pseudomallei* isolation, identification, and antimicrobial susceptibility were conducted in a biosafety II laboratory, following standard biosecurity and institutional safety procedures.

### Case Definition

The cases of melioidotic osteomyelitis and septic arthritis must meet at least 1 of the following 2 criteria: 1. Having the isolation of *B. pseudomallei* strains from bone, marrow, joint fluid, or synovial biopsy, or 2. Having all of the following 4 conditions: ⑴ evidence of osteomyelitis or septic arthritis on direct examination of the bone or joint during a surgical operation, or histopathologic examination or radiographic evidence of infection (e.g., abnormal findings on x-ray, CT scan, MRI, radiolabel scan, ⑵ at least 2 of the following signs or symptoms with no other recognized cause: fever (≧38.8°C), localized swelling, tenderness, heat, or drainage at suspected site of bone infection, or joint pain, swelling, tenderness, heat, evidence of effusion or limitation of motion, ⑶ *B. pseudomallei* cultured from blood, and ⑷ other potential pathogens excluded (Horan et al., 2008; Saavedra-Lozano et al., 2017; Glaudemans et al., 2019; Khiangte et al., 2019). Mortality is determined based on a record of death within the hospitalization days in the routine hospital database (Hantrakun et al., 2019). Furthermore, relapse melioidosis case refers to the patients whose clinical symptoms have been improved with a negative culture after regular anti-melioidosis treatment; however, the development of new symptoms and signs of infection was observed in association with a newly positive *B. pseudomallei* culture, and isolates from the patient (from initial and recurrence) must have identical 16S rRNA and *recA* sequences, and multilocus sequence types (Maharjan et al., 2005; Hayden et al., 2012; Halim I, 2017).

### Epidemiological and Clinical Data

**Figure 1:**
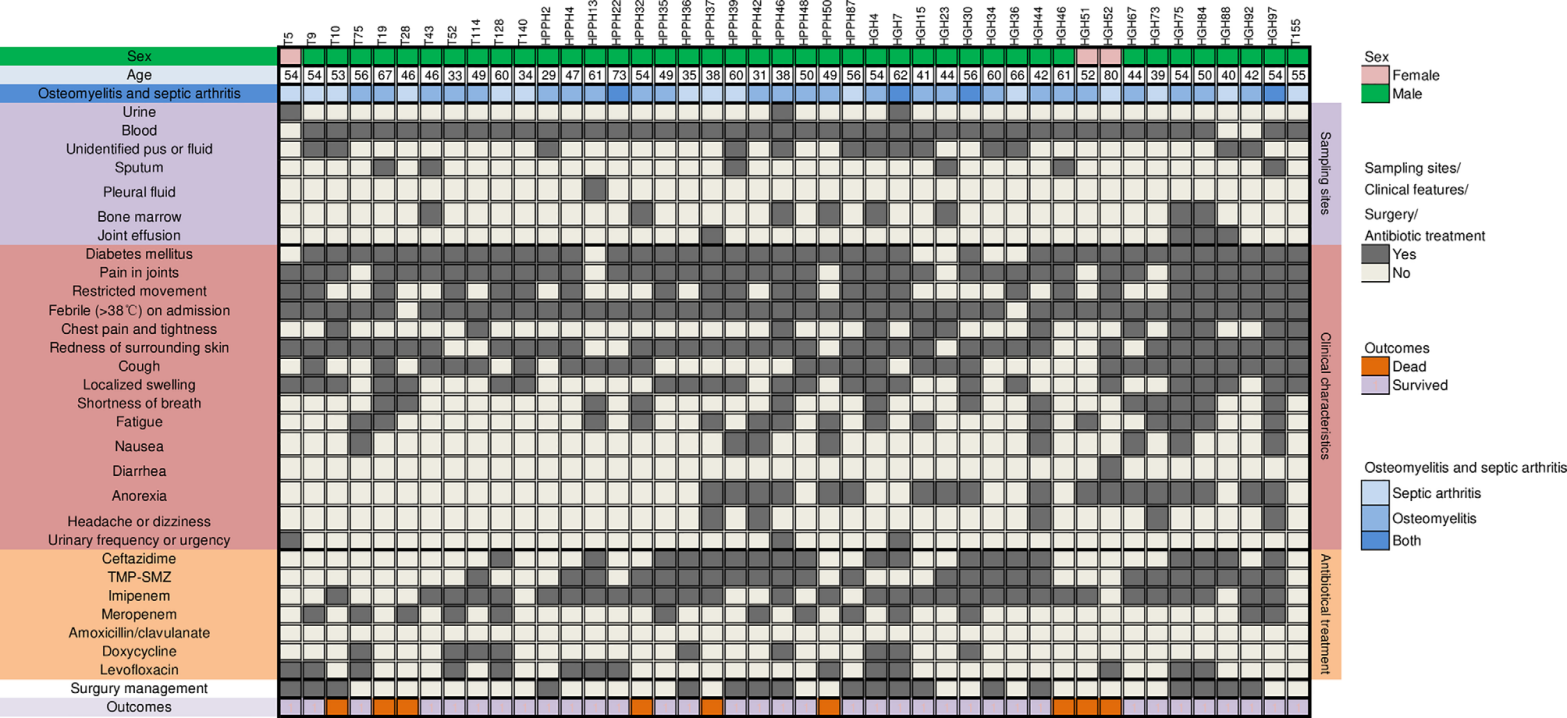
Clinical features and antibiotics treatment in 44 patients having melioidotic osteomyelitis and septic arthritis. trimethoprim/sulfamethoxazole (TMP-SMZ).

During the period from 2010 to 2019, we collected 334 melioidosis patients visiting hospitals in Hainan province, China, of whom 44 (44/334, 13.2%) had melioidotic osteomyelitis and septic arthritis in line with the above criteria. Furthermore, we reviewed their medical reports, which included the following variables: demographic features (age, gender, and occupation), clinical characteristics (symptoms, mortality, and laboratory and imaging results), isolation sites of *B. pseudomallei* strains, potential risk factors (hypertension, diabetes mellitus, alcoholism, and smoking history), and suspected exposure to water and soil. These were detailed in **Table 1** and **Figure 1**, respectively. All these above *B. pseudomallei* strains were forwarded to Department of clinical microbiology of Hainan General Hospital for further confirmation.

**Table 1:** Demographic data of 35 survivors and 9 non-survivors having melioidotic osteomyelitis and septic arthritis.

Parameter	Total (44)	%	Survived (35)	Died (9)	P value
**Demographic features**					
** Gender**					1.000
Male (%)	41	93.2	34	7	
Female (%)	3	6.8	1	2	
** Median age (years)**					0.375
≧65	4	9.1	2	2	
18-65	40	90.9	33	7	
≦18	0		0	0	
**Bone and joint infections**					
Septic arthritis	19	43.2	15	4	1.000
Osteomyelitis	29	65.9	24	5	0.734
**Duration from the onset of symptoms to diagnosis**					0.165
<2 w	24	54.5	22	3	
2w–6m	16	36.4	11	4	
>6 m	4	9.1	2	2	
**Clinical Characteristics**					
Febrile (>38°C) on admission	42	95.5	34	8	0.371
Bacteremia	41	93.2	32	9	1.000
**Predisposing and immunocompromising factors**					
Diabetes mellitus	38	86.4	29	9	0.428
Hazardous alcohol consumption	16	36.4	15	1	0.168
Hypertension	7	15.9	5	2	0.944
Smoking	18	40.9	17	1	0.097
Immunosuppressive medication	1	2.3	1	0	1.000
SLE	1	2.3	1	0	1.000
CKD	6	13.6	5	1	1.000
Anemia	8	18.2	7	1	0.895
Hepatitis and liver cirrhosis	8	18.2	5	3	0.403
Caught cold before onset	2	4.5	1	1	0.371
Postoperative	1	2.3	1	0	1.000
Trauma	7	15.9	6	1	1.000
Tuberculosis	12	27.3	11	1	0.423
Tumor	6	13.6	5	1	1.000
Exposure to soil and dust inhalation	25	56.8	22	3	0.223
Exposed to rain	3	6.8	2	1	0.506
**CT examination**					
Lung infection	35	79.5	27	8	0.752
Abscesses					
Urinary tract	5	11.4	2	3	0.082
Liver	8	18.2	6	2	1.000
Spleen	9	20.5	8	1	0.752
Brain	2	4.5	2	0	1.000
Bone marrow	11	25.0	9	2	1.000
Vessel clot or plaque	2	4.5	1	1	0.371

CKD, Chronic kidney disease; SLE, systemic lupus erythematosus; Immunosuppressive medication; Immunosuppressants or cytotoxic chemotherapy in past 6 months.

### Strain Identification o*f B. pseudomallei*

All *B. pseudomallei* strains collected were streaked onto blood plate agar and incubated at 35°C for 24 h or more if necessary. The fresh colonies were collected and primarily identified based on colony morphology (Raja and Scarsbrook, 2016; Wu et al., 2019; Gassiep et al., 2020) and VITEK Compact 2 (BioMérieux, France) identification cards. Furthermore, the stains were also confirmed through the amplification of the specific 16S rRNA using two universal primers (27F 5’-AGAGTTTGATCCTGGCTCAG-3’ and 1492R 5’-GGTTACCTTGTTACGACTT-3’), and *recA* gene using primers described previously (BUR3, 5’-GA(AG) AAG CAG TTCGGC AA-3’, BUR5 5’-CGATCATGTCGATCGARC-3’) (Payne et al., 2005; Ginther et al., 2015). The sequencing of the 16S rRNA and *recA* was conducted by Ruibiotech (Beijing, China). The consequent comparison of the respective sequences against those in GenBank was performed using online BLASTn (www.ncbi.nlm.nih.gov/blast). BLAST cutoffs for identifying the species were set at 99.0%.

### Statistical Analysis

We evaluated differences in clinical features and demographics between the dead and survival groups with melioidotic osteomyelitis and septic arthritis *via* the Mann-Whitney U test for continuous variables (expressed as the median) and χ^2^ tests for categorical variables, as appropriate. Statistical analyses and data sorting were conducted using GraphPad Prism version 8.0.1. A P value of less than 0.05 was considered statistically significant.

## Results

### Demographic and Clinical Features of Osteomyelitis and Septic Arthritis Melioidosis Cases

A total of 44 patients with melioidotic osteomyelitis and septic arthritis during the study period were reviewed. The average age of these 44 patients was 50.78 ± 11.29 years (range 29-80 years). The gender ratio of male/female was approximately 13.7:1 (41:3). Four patients (10%) were older than 65 years, and none ≦18 years. Of 44 involved cases, 19 patients were farmers who had close contact with contaminated water or soil, 3 were exposed to rain before the disease onset, 7 had a trauma history, and 3 relapsed (Patients 22, 27, and 44, respectively). Interestingly, all relapsed patients had a trauma history.

As shown in **Figure 1**, blood was the most frequent clinical sample where 93.2% (41 cases) patients had bloodstream infections due to *B. pseudomallei*, followed by pus or body fluid (29.5%, 13 cases), bone marrow (18.2%, 8cases). 24 patients (54.5%) have *B. pseudomallei* isolated from multiple sites, as shown in **Table 2**.

**Table 2:** The isolation sites of 44 *Burkholderia pseudomallei* isolates collected from melioidosis patients with osteomyelitis and septic arthritis.

Isolation sites	Total (44)	%	Survived (35)	Died (9)	P value
Blood	41	93.2	32	9	1.000
Unidentified pus or fluid	13	29.5	12	1	0.342
Bone marrow	8	18.2	6	2	1.000
Sputum	6	13.6	4	2	0.766
Joint effusion	4	9.1	3	1	1.000
Urine	3	6.8	3	0	1.000
Pleural fluid	1	2.3	1	0	1.000
Isolation from multiple sites (2 or more)*	24	54.5	18	6	0.657

*defined as one case if B. pseudomallei isolated from pus samples collected from different sites.

### Clinical Features

Of 44 melioidosis BJI cases, diabetes mellitus was the most frequent predisposing factor (38/44, 86.4%), followed by hepatic disorders (8, 18.2%), hypertension (7, 15.9%), and chronic kidney disease (CKD, 6, 13.6%). There were no transplant recipients or HIV patients. Only 1 patient has no underlying status.

The course of the disease before hospitalization ranged from 1 day to over 1 year; most were hospitalized during the first 3 weeks after the onset of symptoms (31/44, 70.5%) (1-9 days, 18 cases; 10-20 days, 13 cases).

**Figure 2:**
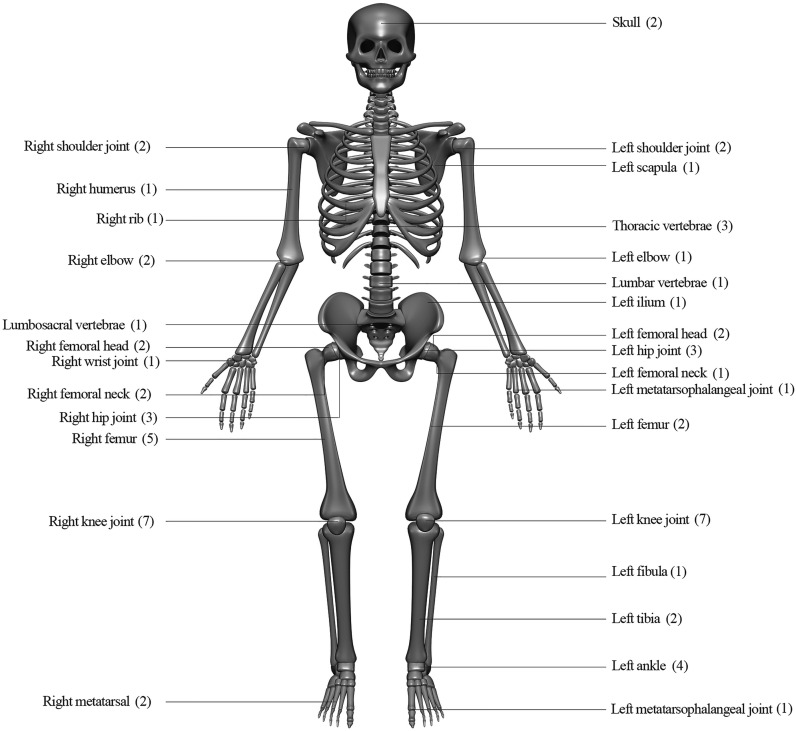
Infection sites and case numbers of 44 patients with melioidotic osteomyelitis and septic arthritis.

With regards to clinical presentations, fever on admission was the most common (42, 95.5%), followed by redness of the surrounding skin (35, 79.5%), cough (26, 59.1%), and joint swelling, and restricted movement (both 24, 54.5%). Furthermore, as in **Figure 2**, knee joints were the most frequently involved bone/joint sites (14 cases), followed by hip joints (6), right femur (5), and ankles (4).

### Antimicrobial Susceptibility, Treatment, and Outcomes

*B. pseudomallei* isolated from these 44 patients were all susceptible to ceftazidime, imipenem and amoxicillin-clavulanic acid, and the susceptible rates to trimethoprim/sulfamethoxazole (TMP-SMZ) were 97.7%, based on the breakpoints set for *B. pseudomallei* in M45 by the Clinical and Laboratory Standards Institute CLSI standard (CLSI, 2015). Data will be published elsewhere. During the study period, information on the treatment regime prescribed was available for 41 patients, of whom 32 received antibiotic combination therapy, and the preferred antibiotic therapy involved imipenem (31), trimethoprim/sulfamethoxazole (TMP-SMZ) (24), ceftazidime (19 patients), levofloxacin (14), meropenem (13), and doxycycline (9). No amoxicillin/clavulanate was administrated.

Moreover, 9 out of 44 patients (20.5%) died. **Table 1** shows the comparison of the detailed clinical features between the survivors (35) and nonsurvivors (9). No differences in clinical features, isolated sites, and CT image results were observed, as shown in **Tables 1** and **2**. In the 9 dead cases, only 1 case received drainage and debridement of the infected sites; by comparison, in 35 survivors, 17 received surgical intervention, showing significant differences.

## Discussion

Melioidosis, an infection due to *B. pseudomallei*, is mainly endemic in northern Australia and parts of southeast Asia (Currie et al., 2010; Gassiep et al., 2020). The ecological niche of *B. pseudomallei* has yet to be confirmed, however, melioidosis is often accessible through an inoculating injury or inhalation of aerosolized bacteria from soil or water (Gassiep et al., 2020). Melioidosis mainly presents as bacteremia and pulmonary diseases, but it is also notorious for its variety of clinical presentations and relapsing nature (Morse et al., 2013; Gassiep et al., 2020). Melioidotic osteomyelitis and septic arthritis are the infrequently-recognized presentation of melioidosis, sometimes also named rheumatological melioidosis (involving one or more of joint, bone, or muscle) (Weerachai Kosuwon, 2003; Teparrakkul, et al., 2008; Morse et al., 2013; Gouse et al., 2017). Its diagnosis requires a combination of history findings, clinical features, and radiological and microbiological confirmation (Raja and Scarsbrook, 2016).

In the present study we retrospectively evaluated 334 melioidosis cases, and 44 (13.2%) melioidotic osteomyelitis and septic arthritis cases were identified. This proportion is close to 14~27% reported in the previous study from northeast Thailand (Weerachai Kosuwon, 2003; Teparrakkul et al., 2008), but higher than 7.6% in a 20-year prospective study from northern Australia, where 41 bone and joint involvement were identified in 536 melioidosis patients, including 27 cases of septic arthritis and 14 cases of osteomyelitis (Morse et al., 2013). This low incidence might also be related to being underdiagnosed due to less awareness of *B. pseudomallei* (Raja and Scarsbrook, 2016).

As previously documented, patients with melioidotic osteomyelitis and septic arthritis had several features consistent with a more chronic course compared with patients without, and a lower mortality rate, but longer fever clearance time and hospitalization as well as a higher rate of relapse (Teparrakkul et al., 2008). Global mortality rates of human melioidosis vary between 9% and 70% (Gassiep et al., 2020). Similarly, as we reported previously, in 159 bacteremic melioidosis patients, 42 died with a mortality rate of 26.4% (Wu et al., 2020), whereas in melioidosis osteomyelitis and septic arthritis patients in the present study, the mortality rate was 20.5% (9/44). Relapse is one of the most important complications of human melioidosis with an incidence ranging from 6% to 23% (McRobb et al., 2014; Li et al., 2017; Perumal Samy et al., 2017). By comparison, in our study, the relapse rate of melioidosis osteomyelitis and septic arthritis was 6.8% (3/44). This might be explained by geographical differences and local medical status. The most commonly presented manifestation was fever, and no specific clinical features can differentiate melioidotic osteomyelitis and septic arthritis from other causative pathogens (Raja and Scarsbrook, 2016). Knee joints were the most frequently invasive sites, as confirmed in previous reports (Raja and Scarsbrook, 2016).

Though melioidosis might present in both immunocompromised and immunocompetent subjects, acquiring melioidosis may depend tremendously on host’s susceptibility, especially in those with predisposing factors such as diabetes mellitus, hypertension, alcohol abuse, tumor, corticosteroid use, or systemic lupus erythematosus (Weerachai Kosuwon, 2003; Raja and Scarsbrook, 2016; Gassiep et al., 2020). In our study, of 44 cases, diabetes was the most common risk factor, and only one patient has no underlying comorbidities. In Thailand, in 7126 culture-confirmed melioidosis cases from 2012 to 2015, the most common comorbidities reported were also diabetes mellitus (43%), followed by hypertension (15%) and CKD (11%) (Hantrakun et al., 2019). By comparison, in India, in 189 culture-proven melioidosis patients, those with diabetes were at a higher risk of musculoskeletal involvement (OR 2.14) (Khiangte et al., 2019). Diabetes was estimated to increase the risk of melioidosis by 100-fold (Raja and Scarsbrook, 2016). Therefore, in the endemic area, the residents and travelers with underlying illnesses should be cautious of melioidosis (Weerachai Kosuwon, 2003; Le Tohic et al., 2019; Gassiep et al., 2020).

A marked male predominance is observed in melioidosis studies (Gassiep et al., 2020; Klimko et al., 2020; Wu et al., 2020). It was infrequently documented that female was over-represented in the bone and joint cohort (Weerachai Kosuwon, 2003). In our study, male patients accounted for 93.2% (41/44). *B. pseudomallei* is distributed in the soil and water; therefore, male predominance is often explained by the fact that males are more likely to come into contact with contaminated soil and water (Raja and Scarsbrook, 2016; Gassiep et al., 2020; Wu et al., 2020).

In the present study, blood was the most frequent clinical sample where *B. pseudomallei* was isolated, accounting for 93.2%, consistent with the recommendation that blood samples should be collected once there was a suspicion of melioidotic osteomyelitis and septic arthritis (Currie et al., 2010; Gouse et al., 2017). Positive blood culture of *B. pseudomallei* together with a radiological abnormality of bones or joints (mainly on CT imaging) strongly suggests this rare disease. Moreover, as proposed by Raja, N. S., needle aspiration from the abscess or bone in the case of osteomyelitis, tissue or bone in discitis, tissue from ulcers or wounds, and bone marrow should also be collected as possible and carefully transported or handled (Raja and Scarsbrook, 2016).

The principle of management of melioidotic bone and joint infections is drainage and extensive debridement of infected sites and appropriate and timely antimicrobial therapy (Dance, 2014; Raja and Scarsbrook, 2016). Repeated wound debridement and removal of necrotic and infected tissue were required (Jayarajah et al., 2020). In our study, 18 patients were managed with surgical management followed by medical treatment, and only 1 died. The antibiotic treatment protocol is initial intensive therapy with high dose intravenous ceftazidime, meropenem or imipenem, followed by clearance therapy with high dose oral TMP-SMZ. In our study, information on the treatment prescribed was available for 41 patients, in whom 32 received antibiotic combination therapy. Imipenem was almost the drug of choice for melioidotic osteomyelitis and septic arthritis, followed by ceftazidime and TMP-SMZ. Doxycycline and levofloxacin were uncommon in our empiric regimens. This is coincident with the administration of the antibiotic in previous documents (Raja and Scarsbrook, 2016). Physicians and laboratories should be aware of that the early recovery of the etiological agent and appropriate antibiotic administration will be helpful for the possible recovery of the patients.

In summary, the present study revealed the demographic features of melioidotic osteomyelitis and septic arthritis. This will be helpful for decision-making in the context of the diagnosis, treatment, and prevention strategies in melioidosis.

## Data Availability Statement

The original contributions presented in the study are included in the article. Further inquiries can be directed to the corresponding author. The data presented in the study are deposited in the GenBank repository, accession number: MZ046382-MZ046425.

## Ethics Statement

The institutional review boards at the Hainan General Hospital approved the study protocol.

## Author Contributions 

HW, XW, XJZ, SC, WM, HH, ZY, SZ, XXZ, and BL collected the clinical and laboratory data. HW and BL made substantial contributions to conception and design, drafted, reviewed, and edited the manuscript. All authors contributed to the article and approved the submitted version.

## Funding

This study was supported by the Key Research and Development Program of Hainan Province, China (Grant No. ZDYF2018113 and ZDYF2019141) to HW and MW, the National Key Research and Development Program of China (Grant Nos. 2018YFC1200100 and 2018YFC1200102) to BL. The funders had no role in study design, data collection, and analysis, decision to publish, or preparation of the manuscript.

## Conflict of Interest

The authors declare that the research was conducted in the absence of any commercial or financial relationships that could be construed as a potential conflict of interest.
